# Blindness in Right Eyes after Enema: A Case of *Klebsiella pneumoniae*-Related Invasive Liver Abscess Syndrome with Endophthalmitis-Caused Blindness as the First Symptom

**DOI:** 10.1155/2024/5573160

**Published:** 2024-02-13

**Authors:** Qi Jin, Xinrui Zhang, Huifen Yang, Bo Zhao, Yubao Wang

**Affiliations:** ^1^Department of Infectious Diseases, Second Hospital of Tianjin Medical University, Tianjin 300211, China; ^2^Respiratory Department, Tianjin Medical University General Hospital, Tianjin 300052, China

## Abstract

We report a case of *Klebsiella pneumoniae* invasive liver abscess syndrome (KPILAS) with endophthalmitis-caused blindness as the first symptom after enema. The patient had diabetes, and his blood glucose was poorly controlled. She developed hematuria after four enemas for cosmetic purposes and later became blind. The eye discharge was cultured, which revealed a *Klebsiella pneumoniae* infection. B ultrasound did not show liver lesions, but computed tomography exhibited abscesses in the right lobe of the liver. Magnetic resonance imaging of the head indicated abscesses. These confirmed the diagnosis of invasive liver abscess syndrome. The patient was given ophthalmic and systemic anti-infection treatments, and her condition was effectively controlled. Unfortunately, the diseased eye still needed to be removed. This case underlines the necessity of avoiding unnecessary risky procedures (such as enemas) in vulnerable populations, the importance of early detection of invasive liver abscess syndrome, and the advantage of computed tomography in detecting liver abscesses.

## 1. Introduction

*Klebsiella pneumoniae* (*Kp*) invasive liver abscess syndrome (KPILAS) is defined as *Kp*-induced liver abscesses complicated by extrahepatic metastatic infection that manifests, as meningitis, brain abscesses, lung abscesses, endophthalmitis, and/or necrotizing fasciitis. [1] Since the 1980s, invasive syndrome resulting from the high viscosity of *Kp* has been frequently reported in East Asia, particularly in Taiwan. [2] As reported, *Kp* infection is the cause of community-acquired meningitis in Taiwan, and liver abscesses have a high mortality rate, up to 30–40%. [3] For ILAS complicated by endophthalmitis, early intervention is warranted to effectively improve the prognosis. [4] The high risk of ILAS is associated with diabetes, malignant tumors of the colon or blood system, inflammatory bowel disease, and neutropenia [5]. In addition, liver abscesses can also be attributed to the invasion of intestinal *Kp* into the liver via the portal venous circulation through bacterial translocation [1]. Furthermore, enema can lead to bacteremia due to bacteria translocation. [5] Klebsiella pneumoniae is a highly virulent pathogen that causes various infections. Moreover, the poor prognosis of invasive liver abscess syndrome portends the need for early treatment. However, early detection of ILAS remains a clinical challenge.

## 2. Case Presentation

A 53-year-old female patient was admitted to the hospital due to “13 days of right eye swelling and pain, 10 days of vision loss, and 1 day of fever.” This patient developed right eye swelling and pain 13 days before admission, with the right-sided headache and no discomforts such as fever, nausea, and vomiting. She visited a local hospital for head computed tomography (CT) and self-reported that the results were not significantly abnormal. Ten days before admission, she noticed decreased visual acuity in the right eye with a yellowish purulent discharge. Therefore, she visited an ophthalmic hospital. Intraocular pressure was 31 mmHg in the right eye and 14 mmHg in the left eye. Ocular ultrasound showed vitreous organic substances in the right eye and vitreous opacity in the left eye. Then, she visited the general hospital for an abdominal ultrasound, which revealed no obvious abnormalities and only multiple polyps in the gallbladder. After that, she visited the ophthalmic hospital again, and 202.90 mg/L C-reactive protein (CRP) was tested. CT results suggested multiple nodules in the right lung and a mass shadow in the right lobe of the liver. In addition, this patient underwent “vitrectomy in the right eye, lens phacoemulsification, and silicone oil injection” seven days before admission. During the surgery, large areas of retinal necrosis were observed in the right eye. After the surgery, the right eye swelling and pain of the patient were aggravated, accompanied by a foreign body sensation and increased yellowish purulent eye discharge. The culture of eye discharge showed *Kp* ++++ (detailed drug sensitivity test results are shown in Table 1). Postoperative blood routine examination exhibited 15.53 × 10^9^/L leukocytes, 90% neutrophils, 122 g/L hemoglobin, 54.00 × 10^9^/L platelets, and 98.47 mg/L CRP. One day before admission, the patient had a fever of up to 37.8°C with chills and vomited once (the vomitus was stomach contents). The routine urine examination during the fever demonstrated glucose 4 +, urinary ketone 1 +, urine occult blood +, and yeast-like fungi +. Later, the patient was admitted to our department for further diagnosis and treatment. This patient has had diabetes for 2 years, but her glycemic control is poor because she does not take “metformin and glibenclamide” regularly.. Over 20 days before admission, she developed gross hematuria without fever and painful, urgent, or frequent urination after four intermittent enemas in the beauty parlor, which were not diagnosed and treated. Hematuria disappeared after approximately 5 days. This patient denied a history of hypertension, coronary heart disease, and cerebrovascular disease.

**Figure 1:**
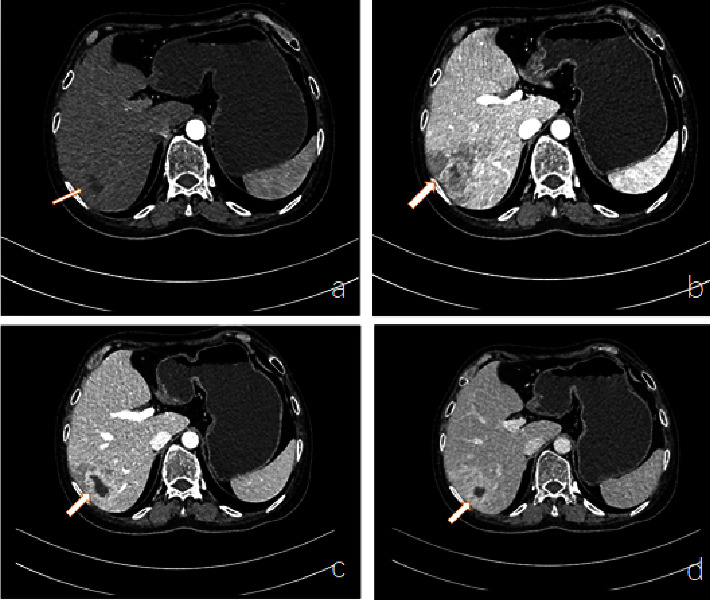
Abdominal enhanced CT on day 5 of admission. On day 5 of admission, an intensive abdominal CT revealed an abscess in the right lobe of the liver. As the arrow indicated, the right posterior lobe of the liver showed a low-density shadow with uneven density; the length was about 3.8 cm, and the septum shadow was visible in the right posterior lobe. Perfusion was enhanced around the lesion, and a strip-like filling defect was shown in the right hepatic vein. Arterial phase (a); venous phase (b); equilibrium phase (c); delay phase (d).

**Figure 2:**
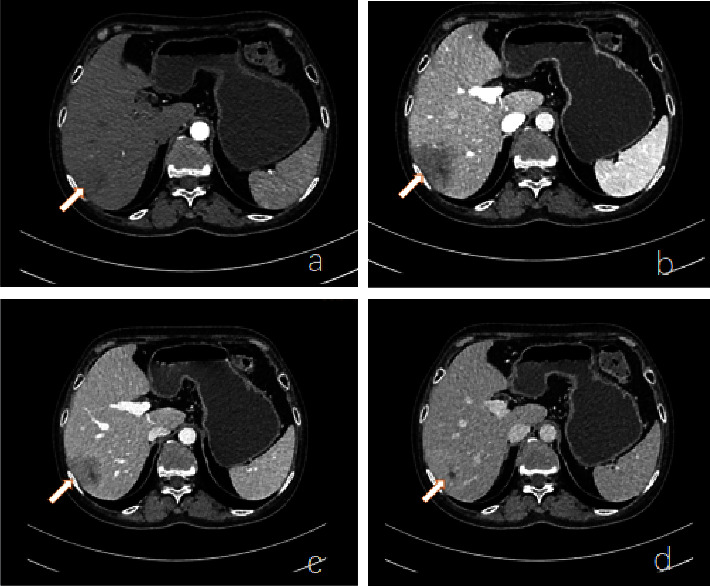
Abdominal enhanced CT on day 16 of admission. On the 16th day of admission, abdominal enhanced CT displayed that the size of the abscess in the right lobe of the liver was 3.4 cm, which was smaller than before. Arterial phase (a); venous phase (b); equilibrium phase (c); delay phase (d).

**Figure 3:**
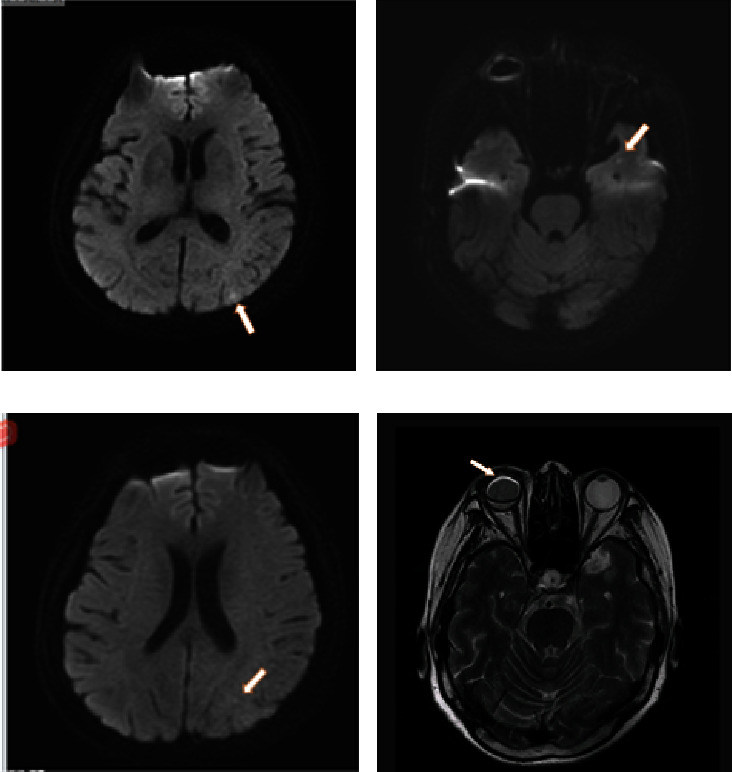
Head/eye MRI on day 18 of admission. On the 18th day of admission, head and eye MRIs were performed. As the arrows indicated, DWI showed slightly high signal intensity in the left occipital lobe (a), left temporal lobe (b), and parietal lobe (c). As the arrow indicated, the signal of the right eyeball was not uniform, and the vitreous body displayed an oval slightly long T1 and T2 signal shadow (d).

After admission, all immune markers were measured, which illustrated 1730.00 mg/dL immunoglobulin G and no abnormalities in other markers. In addition, no obvious abnormality was observed in antineutrophil cytoplasmic antibodies. Two blood cultures both showed no bacterial growth. Fecal culture results were as follows: *Enterococcus faecalis*, 25%; yeast-like fungi, 25%; and *Escherichia coli*, 50%. The results of other tests including routine biochemical test are presented in Table 2. Echocardiography showed no valve excrescences. On the 5th day after admission, the three-phase enhanced CT of the whole abdomen suggested liver abscess and embolus formation of the right hepatic vein (Figure 1). In abdominal color ultrasound, no abnormalities were observed in the liver, gallbladder, pancreas, and spleen. On the 16th day after admission, abdominal enhanced CT displayed that the size of the abscess in the right lobe of the liver was 3.4 cm, which was smaller than before (Figure 2). The magnetic resonance imaging (MRI) of the head displayed multiple mini-brain abscesses (Figure 3). Ocular MRI indicated inflammatory lesions around the right eye ring and changes consistent with postoperative changes in the right eye (Figure 3).

**Figure 4:**
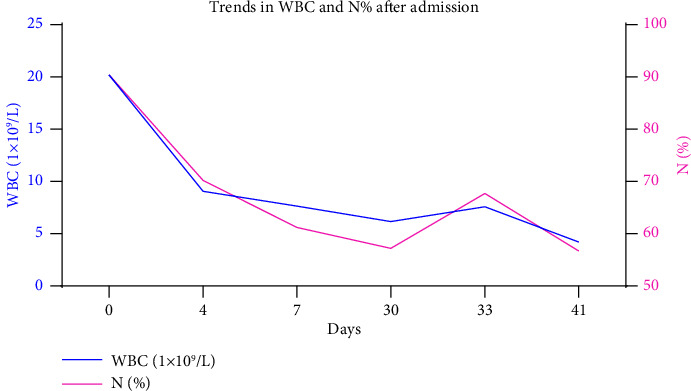
Trend chart of WBC and *N*% after admission. WBC: white cells, *N*%: percentage of neutrophils.

**Figure 5:**
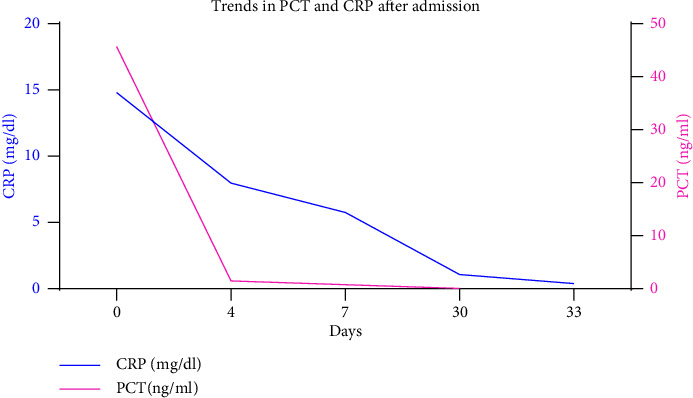
Trends in PCT and CRP after admission. WBC: white cells; *N*%: percentage of neutrophils.

Based on her medical history and laboratory test results, the patient was diagnosed with liver abscess, endophthalmitis (the right eye), and brain abscesses. After admission, the patient was successively intravenously injected with 1 g meropenem (q8 h) for 1 day and with 1 g meropenem (q8 h) combined with 50 mg tigecycline (q12 h; the initial dose was doubled; anti-infection therapy) for 2 days. Nevertheless, the temperature of the patient was poorly controlled; therefore, antibiotic treatment was adjusted. Specifically, she was given intravenous injection of 1 g imipenem/cilastatin (q8 h) combined with 50 mg tigecycline (q12 h) for 5 days and anti-infection treatment of 200 mg etimicin (qd8) combined with 2 g Moxalactam (q12 h) for 17 days, supplemented by symptomatic treatment such as blood sugar control, anticoagulation, and local antibiotics for the eye. After treatment, the temperature and inflammatory indicators, such as hemogram, CRP, and procalcitonin (PCT) of the patient decreased (Figures 4 and 5). The re-examination of the abdomen with enhanced CT revealed that the abscess was smaller than before. The patient was discharged with medicine after her condition improved. The patient had no fever and was generally well. After discharge, the right eyeball was removed in the ophthalmic hospital.

## 3. Discussion

In this case, the patient had poor blood glucose control, an important host factor for ILAS. [6] The gene expression and synthesis of *Kp* capsular polysaccharide and type III pili increase and the *Kp* morphology changes from rodlike to round with high glucose [7] which enhances the resistance of the strain and represses the phagocytosis function of neutrophils. [8] Strict blood glucose control can significantly reduce the extrahepatic metastatic infection of *Kp* in diabetic patients [9]. Enema can cause septicemia in diabetic patients [5]. The patient underwent an enema to promote cosmesis by increasing defecation. She received four enemas before the onset of ILAS and then developed hematuria, which indicated that her intestinal mucosa was damaged and the intestinal contents entered the circulation [10, 11].

Fever is the most common symptom of liver abscess invasion syndrome, while endophthalmitis-caused blindness is relatively rare [6]. In this case, the first symptom was vision loss in the right eye, followed by blindness, and an ophthalmic MRI confirmed endophthalmitis. When the patient visited the hospital, the ophthalmologist considered the possibility of ILAS-induced endogenous endophthalmitis. Nonetheless, liver abscesses were not detected by abdominal B-ultrasound, and CT examination later revealed the abscess focus in the right lobe of the liver, which was the most common location of liver abscess in patients with ILAS [1]. A previous study unveiled that CT had a higher sensitivity (97%) to detect liver abscess than ultrasound (85%) [12]. In the absence of diffuse liver disease, CT is highly specific in excluding liver abscesses and is the most sensitive imaging method for detecting liver abscesses [13].

Endophthalmitis has an incidence rate of about 4–11% in patients with *Kp* liver abscesses, with an extremely poor prognosis, and most patients can develop blindness even after effective anti-infection treatment [1]. For patients with ILAS-induced endophthalmitis, vision could be preserved by anti-infection treatment of the eye within 48 h of the onset [4], suggesting that ILAS-induced endophthalmitis warrants early intervention. In this case, the diseased eyeball of the patient was eventually removed. The late visit of the patient to the ophthalmologist, three days after the occurrence of ocular symptoms, may be the contributor to the failure to retain her vision and eyeballs.

ILAS manifests as a multi-site infection. Therefore, ILAS patients should be thoroughly examined to identify all infection sites. Systemic anti-infection therapy for ILAS should be combined with early use of antibiotics based on drug sensitivity test results. The *Kp* that leads to ILAS decreases the production of extended-spectrum beta-lactamases, with higher sensitivity to third-generation cephalosporins, aminoglycosides, and fluoroquinolones and the highest sensitivity to carbapenems. When reviewing the care of this patient, we found that the course of intravenous antibiotics should have been longer. Typically, this course requires 6–8 weeks for KPILAS. [6] Because the patient was very eager to be discharged, she took the oral medication home and was instructed to follow up at the outpatient clinic. We ought to improve our practice and perform the sufficient intravenous antibiotic treatment course for patients with KPILAS.

This article reports a diabetic patient developing KPILAS with endophthalmitis-caused blindness as the first symptom following enema, which highlights the need to avoid unnecessary risky procedures (such as enema) in vulnerable populations, the importance of early detection of ILAS, and the advantage of CT in detecting liver abscesses.

## Figures and Tables

**Table 1:** Eye secretion culture and antibiotics sensitivity.

Drug sensitivity result: *Klebsiella pneumoniae*++++	
Antibiotics	Sensitivity
Amikacin	S
Ertapenem	S
Ciprofloxacin	S
Meropenem	S
Piperacillin/tazobactam	S
Cefepime	S
Tigecycline	S
Minocycline	R

R, resistant; S, susceptible.

**Table 2:** Laboratory indicators of the patient upon admission.

Parameter	Patient's value	Reference range
Inflammatory index		
CRP, mg/dL	14.8	0–0.8
PCT, ng/mL	45.675	≤0.5
ESR, mm/h	101	0–20
Blood routine examination		
WBC, ×10^9^/L	20.17	3.5–9.5
Neut, %	90.40	40–75
Liver function		
GGT, U/L	118.7	7–45
ALP, U/L	161.60	50–135
ALB, g/L	36.8	40–55
Renal function		
Cr, *μ*mol/L	67	46–92
Blood glucose	11.66	4.1–5.9
HbA1c (%)	8.7	4.5–6.3

CRP: C-reactive protein; PCT: procalcitonin; ESR: erythrocyte sedimentation; WBC: leukocyte; Neut %: neutrophil percentage; Glu: glucose.

## Data Availability

The data used to support the findings of this study are included within the paper.
